# Effects of Three Extraction Methods on Avocado Oil Lipid Compounds Analyzed via UPLC-TOF-MS/MS with OPLS-DA

**DOI:** 10.3390/foods12061174

**Published:** 2023-03-10

**Authors:** Yijun Liu, Qiuyu Xia, Yangyang Qian, Yu Kuang, Jiameng Liu, Lijing Lin

**Affiliations:** 1 Hainan Key Laboratory of Storage & Processing of Fruits and Vegetables, Agricultural Products Processing Research Institute, Chinese Academy of Tropical Agricultural Sciences, Zhanjiang 524001, China; liuyijun-1@163.com (Y.L.);; 2 College of Food Science and Technology, Guangdong Ocean University, Zhanjiang 524088, China; 3 College of Tea (Pu’er), West Yunnan University of Applied Sciences, Pu’er 665000, China; 4 Yunnan Dehong Insititution of Tropical Agricultural Science, Ruili 678600, China; 5 Key Laboratory of Tropical Crop Products Processing of the Ministry of Agriculture and Rural Affairs, Agricultural Products Processing Research Institute, Chinese Academy of Tropical Agricultural Sciences, Zhanjiang 524001, China

**Keywords:** avocado oil, lipids, UPLC-TOF-MS/MS, extraction methods

## Abstract

Avocado oil is excellent functional oil. Effects of three extraction methods (squeezing extraction, supercritical carbon dioxide extraction, and aqueous extraction) on the species, composition, and contents of lipids in avocado oil were analyzed via ultra-performance liquid chromatography–time-of-flight tandem mass spectrometry (UPLC-TOF-MS/MS), and the differential components of lipids were revealed by OrthogonalPartialLeast Squares-DiscriminantAnalysis (OPLS-DA), S-plot combined with variable importance in the projection (VIP). The results showed that the fatty acid composition of avocado oil mainly consisted of oleic acid (36–42%), palmitic acid (25–26%), linoleic acid (14–18%), and palmitoleic acid (10–12%). A total of 134 lipids were identified first from avocado oil, including 122 glycerides and 12 phospholipids, and the total number of carbon atoms contained in the fatty acid side chains of the lipids was 32–68, and the number of double bonds was 0–9. Forty-eight differential lipid compounds with significant effects of the three extraction methods on the lipid composition of avocado oil were excavated, among which the differences in triglycerides (TG), phosphatidylethanol (PEtOH), and phosphatidylmethanol (PMeOH) contents were highly significant, which provided basic data to support the subsequent guidance of avocado oil processing, quality evaluation, and functional studies.

## 1. Introduction

Avocado is typical subtropical fruit, and “Hass”, “Choquette”, “Gwen”, “Lula” and “Maluma” are the main cultivated varieties, among which “Hass” has the largest planting area. Avocado is rich in nutrients, containing a variety of vitamins, tocopherols, and trace metal elements such as calcium, magnesium, and zinc, and the oil in the avocado pulp mainly consists of various monounsaturated fatty acids and polyunsaturated fatty acids, of which oleic acid accounts for 34% to 81%, 7.2–38.9% for palmitic acid, 6–26.6% for linoleic acid, 2.1–5.8% for linolenic acid, etc. [1,2,3]. Avocado oil has been applied to the development of products that aid in lowering blood pressure, are anti-inflammatory, and promote wound healing, with promising applications [4].

Fatty acids are very important components of avocado oil, and their mechanism of action enhances vascular function, reduces the deterioration of nephropathy, and improves nonalcoholic fatty liver in hypertensive rats by improving mitochondrial dysfunction, reducing mitochondrial oxidative stress, decreasing reactive nitrogen species (RNS) production and normalizing NOx activity [5,6,7]. Cristian et al. [8] used avocado oil instillation in hypertensive rats and reduced diastolic and systolic blood pressure by 21.2% and 15.5%, respectively. In addition, avocado oil not only increases collagen synthesis, reduced the number of inflammatory cells, accelerated the coagulation process and the regeneration of epithelial cells, thus accelerating wound healing [9], but also regulated brain-derived neurotrophic factor (BDNF), oxidative stress and apoptotic molecules, and protected SH–SY5Y cells against cortisol-induced cytotoxicity [10]. Omar et al. [11] used rats as a model for type 2 diabetes and confirmed that lipid components such as oleic acid in avocado oil delayed the development of diabetic nephropathy. Pham et al. [12] isolated DKB122 from avocado oil extract, which effectively inhibited TNF-α or LPS-induced p65 nuclear migration in HEI-OC1 cells and THP-1 cells and reduced TNF-α-induced expression of inflammatory chemokines and interleukin genes.

The functionality of avocado oil was closely related to its nutritional composition, while the nutritional quality of avocado oil was influenced by factors such as fruit variety [3,13], extraction method [14], and fruit storage method [15]. Lozano et al. [13] confirmed that total sterols were higher in immature fruits (1.1–6.2%) than in mature fruits (0.8–2.0%) in four avocado varieties, “Zutano”, “Bacon”, “Fuerte”, and “Lula”. Ultrasonic-assisted water extraction [16], mechanical pressing [17], and supercritical CO_2_ extraction [18,19,20] methods were commonly used to extract avocado, and the results of a comparative study by Tan et al. [18,19,20] showed that different extraction methods had an effect on physicochemical properties such as iodine value in avocado oil, but did not have a large effect on fatty acid composition such as oleic acid, which varied in content. Fernanda et al. [17] showed that drying of avocado at 60 °C combined with mechanical pressing resulted in better retention of the biological activity of avocado oil. The drying method and storage conditions had a greater influence on the quality of avocado oil. Chaiyavat et al. [21] showed that drying conditions at 80 °C and above had a significant effect on the stability of avocado oil and that light-free conditions helped to extend the shelf life of avocado oil. The stability and quality of avocado oil were susceptible to temperature effects and were not suitable for continuous heating processes [22,23]. However, little research had been reported on the effects of extraction methods on avocado lipid composition. In this study, ultra-performance liquid chromatography–time-of-flight tandem mass spectrometry (UPLC-TOF-MS/MS) was used to investigate the effects of extraction methods on the components of avocado oil quality, to explore the differential components of avocado oil quality by extraction methods, and to provide basic data support for avocado oil extraction methods, product development, and functional studies.

## 2. Materials and Methods

### 2.1. Preparation of Avocado Oil

The variety of avocado was “Hass”, purchased from Zhanjiang Chang-da-Chang Super Shopping Plaza Co., and the fruit was 80% mature (skin color changed from dark green to dark brown). Referring to the method of Liu et al. [1], three methods of squeezing extraction, supercritical carbon dioxide extraction, and aqueous extraction were used to extract the oil from avocado pulp, the crude oil was centrifuged in a centrifuge at 5000× *g* for 10 min, and the collected oil layer was stored at 4 °C. The parameters of that three methods were as follows:

Squeezing extraction: Avocado pulp dried at 55 °C for 24 h was squeezed by the sing screw expeller with normal temperature mode, and the crude oils were collected and centrifuged at 5000× *g* for 10 min, and the crude oil layer was collected.

Supercritical carbon dioxide extraction: Avocado pulp dried at 55 °C for 24 h was extracted in a supercritical carbon dioxide extractor. The extraction temperature grades I and II were 45 °C and 50 °C, respectively, and the extraction pressure grades I and II were 5 MPa and 6 MPa, respectively, and the crude oil was collected.

Aqueous extraction: A 1 kg sample of avocado oils and 2 kg distilled water were beaten and mixed evenly, and then colloid mill was used for 1 min to obtain slurry solution. Then, 2 kg distilled water was used to clean the machine, and cleaning solutions were collected. The slurry solution and cleaning solution, adjusted to 8.0 with a 1.00 mol/L sodium hydroxide solution, were combined and stirred for 1.5 h at 75 °C water bath, then the mixed solution was centrifuged at 25,000× *g* for 10 min, and the upper crude oil was collected.

### 2.2. Instrumentations

Squeezer (OP101, Shenzhen Yimeikang Electronic Commerce Co., Ltd., Shenzhen, China), supercritical carbon dioxide extractor (HSFE-5 + 1, Jiangsu Gaoke Pharmaceutical Equipment Co., Ltd., Nantong, China), high-speed freezing centrifuge (CR22GIII, Hitachi Limited, Tokyo, Japan), juicer (JYL-C020E, Jiuyang Co., Ltd., Jinan, China), pipeline high shear colloid mill (ZVF300-G5R5/P7R5T4MD, Shanghai Qike Machinery Equipment Co., Ltd., Shanghai, China), ultraviolet–visible spectrophotometer (UV-1780, Shimadzu Corporation, Kyoto, Japan), gas chromatography–mass spectrometry (AOC5000-GC/MS-QP2010plus, Shimadzu Corporation, Kyoto, Japan), ultra-high performance liquid chromatograph–time-of-flight tandem mass spectrometer (LC-30A liquid chromatography, Shimadzu Corporation, Kyoto, Japan), ultra-pure water system (Milli-Q-Synthesis, Milli-pore Company, Boston, MA, USA), multi-tube vortex mixer (MTV-100, Hangzhou Aosheng Instrument Co., Ltd., Hangzhou, China), nitrogen blower (DC-24, Shanghai Ampu Experimental Technology Co., Ltd., Shanghai, China).

### 2.3. Determination of Fatty Acid Composition

The fatty acid composition in avocado oil was determined by potassium hydroxide methylation method with reference to the method of Liu et al. [24]. A sample of 1.0 μL passed through the chromatographic column (DB-FastFA, 30 m × 0.25 mm × 0.25 μm, Agilent, California, USA) in gas chromatography–mass spectrometry with the inlet temperature of 260 °C, nitrogen as the carrier gas, and the split ratio of 20:1. The initial temperature of the column was 150 °C, then it was raised below the program, and the speed of 10 C/min was raised to 210 °C and kept for 8 min, and the speed of 20 °C/min was raised to 230 °C and kept for 6 min. Finally, the sample passed through a detector with a temperature of 280 °C.

### 2.4. Determination of Lipid Composition

The lipid composition in avocado oil was determined equipped with a Phenomenex Kinete C18 column (100 × 2.1 mm, 2.6 µm, Phenomenex, Torrance, CA, USA) with reference to the method of Liu et al. [24,25]. One microliter of sample was pumped onto the C18 column at a rate of 0.4 mL/min. The column temperature and chamber temperature were 60 °C and 4 °C, respectively. The mobile phases A and B consisted of H_2_O–methanol–acetonitrile = 1:1:1 (containing 5 mmol/L ammonium acetate) and isopropanol-acetonitrile = 5:1 (containing 5 mmol/L ammonium acetate). The elution program of mobile phase was performed as 20% B for 0.5 min, 40% B for 1.5 min, 60% B for 3 min, 98% B for 13 min, 20% B for 13 min, and 20%B for 17 min.

### 2.5. Data Processing and Analysis

All samples were measured 3 times in parallel. The qualitative analysis of shotgun-MS data was treated by the LipidView software (v2.0, ABSciex, Concord, ON, Canada). In the process of data analysis, the analysis parameters were set according to the following figures: the mass tolerance was 0.5, the minimum signal-to-noise ratio was 10, the minimum% intensity was 1, the average flow injection spectrum from the top was 30% TIC, and the total double bonds were ≤12. OriginPro (2021, OriginLab Corporation, Northampton, UK) was used for plotting, thermal map analysis, and statistical analysis, and the SIMCA (14.1, Sartorius Lab Instruments GmbH & Co., KG, Goettingen, Germany) was used for PCA, OPLS-DA, VIP, and S-plot analysis, etc.

## 3. Results and Analysis

### 3.1. Analysis of Fatty Acid Composition and Lipid Composition in Avocado Oil

The fatty acid composition in avocado oil was determined by gas chromatography-mass spectrometer (GC-MS), and the retention time of each fatty acid standard was characterized with reference to the retention time of each fatty acid standard, and the relative percentage content was calculated according to the normalization method of chromatographic peak area. The fatty acids of avocado oil mainly consisted of oleic acid (36–42%), palmitic acid (25–26%), linoleic acid (14–18%), palmitoleic acid (10–12%), isoleic acid (6–7%), linolenic acid (0.5–0.8%) and stearic acid (0.5–0.6%). The content of saturated fatty acids and unsaturated fatty acids in avocado oil obtained by three extraction methods was about 26% and 73%, among which the content of monounsaturated fatty acids ranged from 54 to 60%.

**Figure 1:**
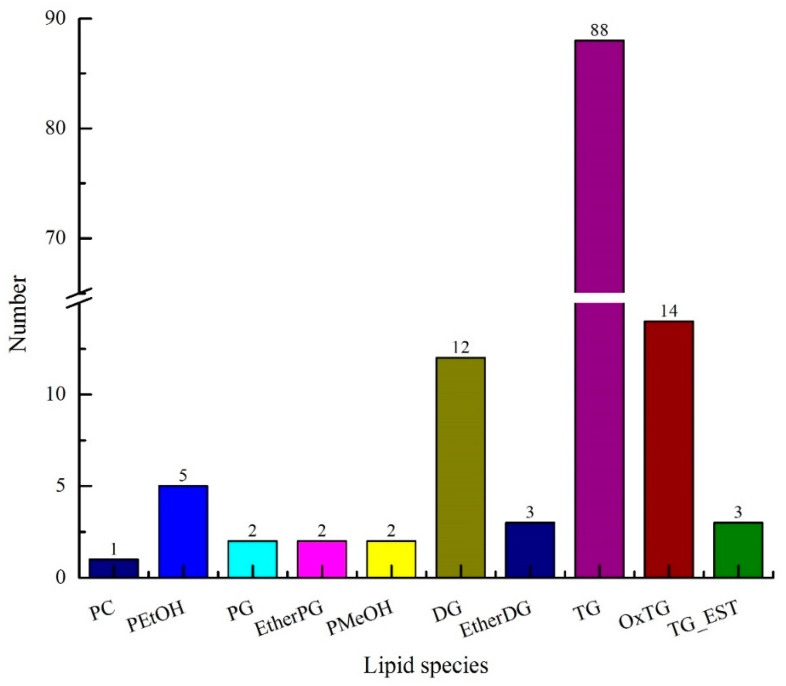
Lipids of composition of avocado oil.

UPLC-TOF-MS/MS combined with composite scanning mode was used to analyze the lipid composition in avocado oil, as well as the accurate relative molecular weight, isotope distribution, and secondary mass spectrometry fragmentation information. As shown in Figure 1, a total of 134 lipids were identified in avocado oil, including 122 glycerides and 12 phospholipids. Glycerides were composed of diacylglycerol (DG), ether-linked diacylglycerol (EtherDG), triglycerides (TG), oxidized triglycerides (OxTG), triglycerides (TG_EST), and ether-linked triglycerides (EtherTG), and among of which type numbers were 12, 3, 88, 14, 3, and 2, respectively. Phospholipids were composed of phosphatidylcholine (PC), phosphatidylethanol (PEtOH), phosphatidylglycerol (PG), ether-linked phosphatidylglycerol (EtherPG), and phosphatidylmethanol (PMeOH), and among of which, type numbers were 1, 5, 2, 2, and 2, respectively.

As can be seen from Table 1, the total number of carbon atoms in the fatty acid side chains of lipids in avocado oil was 32–68, and the number of double bonds was 0–9. The carbon atoms and double bonds number of DG, EtherDG, TG, OxTG, TG_EST, and EtherTG in glycerides were (32–42, 0–5), (34–36, 2–4), (34–64, 0–9), (50–54, 2–5), (66–68, 3–4), and (53–55, 2–5), respectively. The side chain of glycerides was mainly composed of C15, C16, C17, C18, and C19. The carbon atoms and double bonds number of PC, PEtOH, PG, EtherPG, and PMeOH in glycerides were (34, 2), (34–36, 1–4), (32–34, 0–1), (34–37, 3–5), and (34, 0), respectively.

### 3.2. Analysis of Lipid Content in Avocado Oil

**Figure 2:**
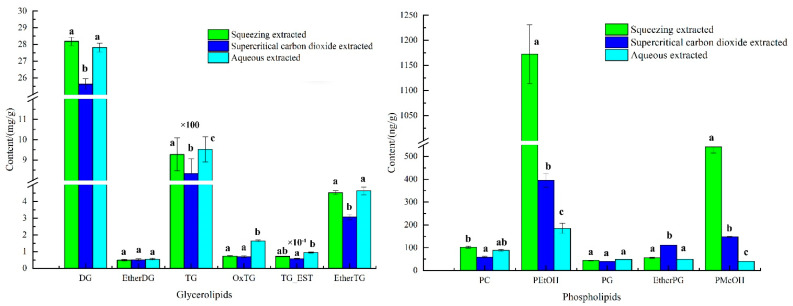
The composed of phospholipids and glycerides in avocado oil from three extraction methods. Note: Different letters a, b and c represented significant difference.

The lipid content of avocado oil obtained by three extraction methods was shown in Figure 2. As shown in Figure 2, the highest TG content in glycerides of avocado oil was (830–960) mg/g, followed by DG at (25–30) mg/g, and the highest PEtOH content in phospholipids was (180–1200) ng/g, followed by PMeOH at (40–545) ng/g. The significant difference results showed that the three extraction methods had the highest effect on the TG, PEtOH and PMeOH contents were highly significant, and the differences for EtherDG and PG contents were not significant.

### 3.3. Modeling and Evaluation of Differential Metabolites of Lipids in Avocado Oil

**Figure 3:**
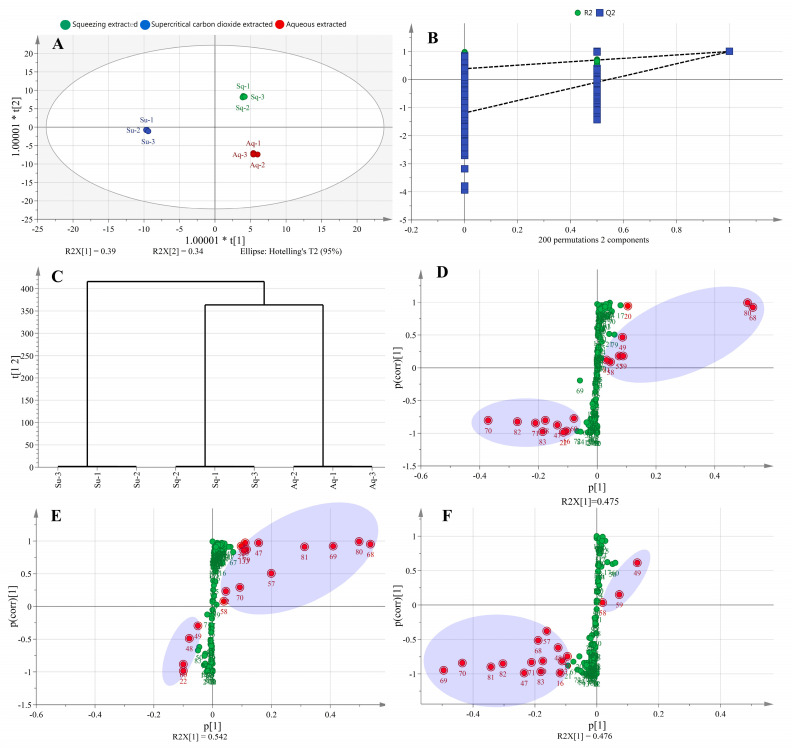
Scores plot of OPLS-DA model (**A**), cross-validation of OPLS-DA model (**B**), HCA (**C**), and S-plot of lipids with different extraction methods. Sq, Su, Aq in Figure 3A represent squeezing extraction, supercritical carbon dioxide extraction, and aqueous extraction, respectively. (**D**) Represents S–plot of squeezing extracted and aqueous extracted, (**E**) represents S–plot of supercritical carbon dioxide extracted and aqueous extracted, and (**F**) represents S–plot of supercritical carbon dioxide extracted and squeezing extracted. The red dots and green dots in Figure 3D–F indicate metabolites with VIP values > 1, and VIP values < 1, respectively.

From Figure 3A, it can be seen that the avocado oil samples obtained by the three extraction methods could be better distinguished in the OPLS–DA model, and the three oil samples were distributed in the first, third, and fourth quadrants, indicating that they differed from each other. From Figure 3B, it can be seen that the lipid composition data obtained by the three extraction methods were subjected to permutation test and cross–validation analysis (CV–ANOVA), the intercepts of R^2^ and Q^2^ curves with vertical coordinates were less than one, and the intercept of Q^2^ in vertical coordinates was less than zero, indicating that the established OPLS–DA model did not show any overfitting phenomenon. In addition, the significance probability value *p* < 0.05 in CV–ANOVA analysis indicated that the established OPLS–DA model was stable, reliable, and statistically significant [26]. As shown in Figure 3C, the avocado oil obtained from the three extraction methods was well clustered.

The S–plot was used to identify significant differential metabolites between the two samples, and metabolites with large contributions were concentrated at two ends of the S–plot, while those with small contributions were concentrated around the origin [27]. The abscissa and ordinate represented the co-correlation coefficient and correlation coefficient of the principal component and metabolite, respectively. The red dots in Figure 3D–F indicate metabolites with VIP values >1. From Figure 3D, seventeen significantly different components were analyzed between the squeezing extraction and aqueous extraction methods, including eight metabolites with VIP values >2, namely TG 52:2|TG 16:0_18:1_18:1 (68), TG 54:3|TG 18:1_18:1_18:1 (80), TG 52:4|TG 16:1_18:1_18:2 (70), TG 50:2|TG 16:0_16:1_18:1 (58), TG 54:5|TG 18:1_18:2_18:2 (82), TG 50:3|TG 16:0_16:1_18:2 (59), TG 50:1|TG 16:0_16:0_18:1 (57), TG 52:5|TG 16:1_18:2_18:2 (71). From Figure 3 (E), eighteen significantly different components were analyzed between the supercritical carbon dioxide extraction and aqueous extraction methods, including eight metabolites with VIP values >2, namely TG 52:2|TG 16:0_18:1_18:1 (68), TG 54:3|TG 18:1_18:1_18:1 (80), TG 52:3|TG 16:0_18:1_18:2 (69), TG 54:4|TG 18:1_18:1_18:2 (81), TG 50:2|TG 16:0_16:1_18:1 (58), TG 50:1|TG 16:0_16:0_18:1 (57), TG 50:3|TG 16:0_16:1_18:2 (59), TG 52:4|TG 16:1_18:1_18:2 (70). From Figure 3F, seventeen significantly different components were analyzed between the supercritical carbon dioxide extraction and squeezing extraction methods, including ten metabolites with VIP values >2, namely TG 52:3|TG 16:0_18:1_18:2 (69), TG 52:4|TG 16:1_18:1_18:2 (70), TG 54:4|TG 18:1_18:1_18:2 (81), TG 54:5|TG 18:1_18:2_18:2 (82), TG 50:3|TG 16:0_16:1_18:2 (59), TG 50:1|TG 16:0_16:0_18:1 (57), TG 48:0|TG 16:0_16:0_16:0 (47), TG 50:2|TG 16:0_16:1_18:1 (58), TG 52:2|TG 16:0_18:1_18:1 (68), TG 52:5|TG 16:1_18:2_18:2 (71).

### 3.4. Differential Metabolite Differential Analysis and Mass Spectrometry of Lipids in Avocado Oil

**Figure 4:**
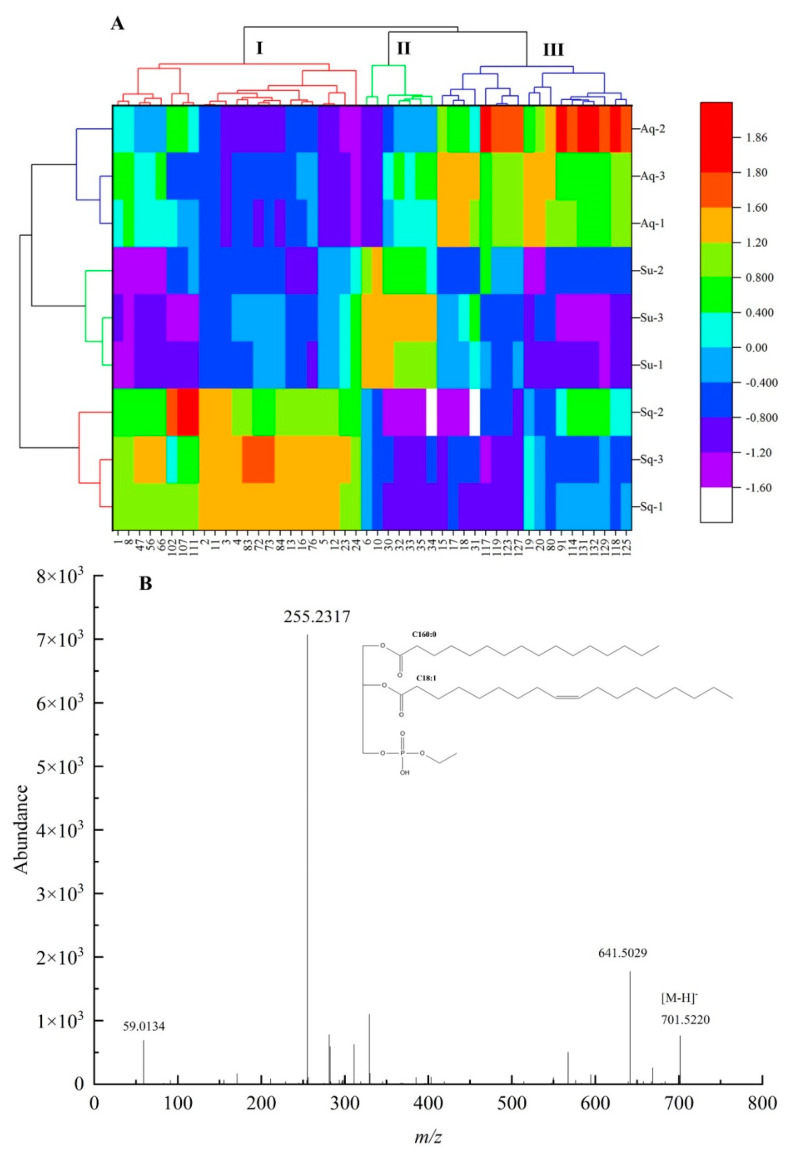
(**A**) Represented the thermogram of lipid components with significant differences among the three extraction methods, and (**B**) represented the mass spectrogram of PETOH 34:1|PETOH 16:0 _18:1 in negativity mode.

VIP analysis of lipid components in avocado oil obtained by the three extraction methods was performed on the OPLS–DA model, 77 differential metabolites with VIP value > 1 were obtained, and Krural–Walli’s significance test was performed on the 77 metabolites, and 48 significantly different metabolites were obtained. The differential metabolites were subjected to Z–score transformation to standardize the data, Z–score = (original data – mean)/standard deviation, and the standardized data were produced as a heat map, as shown in Figure 4A.

From Figure 4A, it can be seen that the metabolites of the three extraction methods can be categorized into three groups. Groups I, II, and III were the groups with significant upregulation of differential lipid components obtained by the squeezing extraction method, supercritical carbon dioxide extraction method, and aqueous extraction method, respectively, in which 23 lipid components including PC 34:2 (1), PEtOH 34:1|PEtOH 16:0_18:1 (2), PEtOH 34:2|PEtOH 16:0_18:2 (3), PEtOH 36:2|PEtOH 18:1_18:1 (4), TG 60:3|TG 24:0_18:1_18:2/TG 26:0_16:1_18:2 (102), and TG 61:4|TG 25:0_18:2_18:2 (107) were upregulated in group I. Seven lipid components including PEtOH 36:4|PEtOH 18:2_18:2 (6), PG O–37:5|PG O–16:2_21:3 (10), TG 38:0|TG 8:0_14:0_16:0 (30), TG 40:0|TG 10:0_14:0_16:0 (32), TG 40:1|TG 10:0_12:0_18:1 (33), TG 42:1|TG 8:0_16:0_18:1 (35) were upregulated in group II. Twenty–three lipid components including TG 54:3|TG 18:1_18:1_18:1 (80), TG 59:1|TG 16:0_25:0_18:1 (97), TG 64:2|TG 28:0_18:1_18:1 (114), TG 50:3;1O|TG 16:0_18:2_16:1;1O (117), TG 52:2;1O|TG 16:0_18:1_18:1;1O (118), TG 52:3;1O|TG 16:0_18:1_18:2;1O (119), TG 52:2;2O|TG 16:0_19:1_17:1;2O (123), TG 52:4;2O|TG 16:0_18:2_18:2;2O (125), TG 54:3;2O|TG 18:1_18:1_18:1;2O (127), TG 54:5;2O|TG 18:2_19:2_17:1;2O (129) were upregulated in group III.

After the precursor ions of selected lipid molecules enter the mass spectrometry Q^2^, collision–induced dissociation (CID) occurs at a certain collision energy (CE), resulting in fragment ions, and the neutral loss of specific fragment ions or specific functional groups from lipid molecules lead to diagnostic ions. In this study, the differential metabolite PEtOH 34:1|PEtOH 16:0_18:1 in phosphatidylethanol (PEtOH) was used as an example to analyze its mass spectrometric behavior and fracture mechanism in detail, as shown in Figure 4B. From Figure 4B, *m*/*z* 701.5220 corresponded to the mass spectrum information of [M − H]− parent ion of PEtOH 34:1, *m*/*z* 125.0009 was phosphoethanol, and *m*/*z* 255.2327 and *m*/*z* 281.2475 represented the mass spectrum information of [FA 16:0–H]− and [FA 18:1–H]−, respectively.

## 4. Discussion

There were few studies on the lipid composition in avocado oil, but there were more studies on pitaya seed oil, coffee bean oil, canola oil, and soybean oil. The present study showed that avocado oil was mainly composed of oleic acid (36–42%), palmitic acid (25–26%), linoleic acid (14–18%), and palmitoleic acid (10–12%), similar to the fatty acid composition in avocado reported by Fernandes et al. [28], but there was variability in the fatty acid content, such as low oleic acid content of 10–20%, palmitic acid content was 10–15% higher, and linoleic acid was about 5% higher, with differences in the variety and origin of avocado leading to differences between the two.

In this study, a total of 134 lipid molecules were identified from different extraction methods, which was less than that of pitaya seed oil (152) [24] and cycad oil (169) [29]. Avocado oil was similar to pitaya seed oil in that it consists mainly of glycerides and phospholipids and had the highest content of TG in glycerides and PEtOH in phospholipids, but some variability exists in that avocado oil contained phosphatidylcholine PC, which was lacking in dragon fruit seed oil [24]. Additionally, it was based on the variability of glycerides and phospholipid species in oils and fats that much research work had been completed to identify the source, quality, and variety of oils and fats. Tian et al. [30] analyzed and identified 24 triglycerides, mainly OOO (triglyceride of trioleic acid), OOL (triglyceride of 1,2–dioleic acid–3–linoleic acid), OOP (triglyceride of 1,2–dioleic acid–3–palmitic acid), and other unsaturated triglycerides from six different oil tea species and nine different common oil tea varieties and constructed a fingerprint profile of triglycerides in oil tea seeds. The fingerprint profiles of triglycerides in oil tea seeds were also constructed to identify different varieties of oil tea seed oil. The results of Zhao et al. [31] showed that LL and OO in DAGs and OLLn and LLL in TAGs were important indicators for the grade identification of olive oil, and these indicators could be used for the quality identification of different grades of olive oil. Therefore, the information on the type and content of microscopic lipid components in oils and fats by profiling could provide new ideas and more accurate analysis for the source, type, and quality identification of oils and fats.

The fatty acid content and composition of avocado oil varied depending on the variety, origin [32], and extraction method [1,19,33], with the differences existing in extraction methods being particularly pronounced, yet there were few comparative studies from a microscopic perspective. In this study, 48 differential metabolites were identified from 134 lipid components using OPLS–DA combined with VIP and other methods, among which 23, 7, and 23 differential metabolites were upregulated by the squeezing extraction, supercritical carbon dioxide extraction, and aqueous extraction, respectively, while phospholipids were more abundant in avocado oil obtained by supercritical carbon dioxide extraction, which was in accordance with the principle of similar compatibility. The long extraction process by the aqueous extraction method and the long air contact time resulted in higher OxTG content. In addition, the principles of the pressing method and extraction method were different, resulting in differences in both PMeOH and glycerol ester compounds. Therefore, revealing the differences among the oils and fats obtained by the three extraction methods from the perspective of lipid molecules could provide basic data to support the study of the transformation mechanism of lipid molecules during processing.

## 5. Conclusions

In this study, the UPLC–TOF–MS/MS was used to profile the lipid profile of avocado oil first, and 134 lipid components were identified, including 122 glycerides and 12 phospholipids. The total number of carbon atoms contained in the fatty acid side chains of the lipids ranged from 32 to 68, and the number of double bonds ranged from 0 to 9. The differences between the three extraction methods were highly significant for the contents of TG, PEtOH, and PMeOH, and not significant for the contents of EtherDG and PG. The analysis by OPLS–DA, S–plot, and VIP identified 44 differential metabolic components, which provided theoretical data for guiding the avocado oil’s processing, quality evaluation, and in–depth functional research.

## Figures and Tables

**Table 1:** Composition of the 134 lipids in avocado oil.

No.	Average Rt (min)	Average Mz	Lipid Name	Adduct Type	Formula	Ontology
1	5.12	758.56958	PC 34:2	[M − H]−	C_39_H_75_O_8_P	PC
2	4.089	701.50745	PEtOH 34:1|PEtOH 16:0_18:1	[M − H]−	C_39_H_73_O_8_P	PEtOH
3	3.864	699.50714	PEtOH 34:2|PEtOH 16:0_18:2	[M − H]−	C_41_H_77_O_8_P	PEtOH
4	4.114	727.52087	PEtOH 36:2|PEtOH 18:1_18:1	[M − H]−	C_41_H_75_O_8_P	PEtOH
5	3.76	725.51746	PEtOH 36:3|PEtOH 18:1_18:2	[M − H]−	C_41_H_73_O_8_P	PEtOH
6	3.493	723.49786	PEtOH 36:4|PEtOH 18:2_18:2	[M − H]−	C_38_H_75_O_10_P	PEtOH
7	3.834	721.5014	PG 32:0|PG 16:0_16:0	[M − H]−	C_40_H_77_O_10_P	PG
8	3.939	747.51971	PG 34:1|PG 16:0_18:1	[M − H]−	C_40_H_75_O_9_P	PG
9	4.044	729.50385	PG O–34:3|PG O–18:3_16:0	[M − H]−	C_43_H_77_O_9_P	EtherPG
10	3.944	767.52594	PG O–37:5|PG O–16:2_21:3	[M − H]−	C_38_H_73_O_8_P	EtherPG
11	4.029	687.49158	PMeOH 34:1|PMeOH 16:0_18:1	[M − H]−	C_38_H_71_O_8_P	PMeOH
12	3.819	685.48138	PMeOH 34:2|PMeOH 16:0_18:2	[M − H]−	C_38_H_71_O_8_P	PMeOH
13	5.982	586.52954	DG 32:0|DG 16:0_16:0	[M + NH4]+	C_35_H_68_O_5_	DG
14	5.529	584.51715	DG 32:1|DG 16:0_16:1	[M + NH4]+	C_35_H_66_O_5_	DG
15	5.117	582.49945	DG 32:2|DG 16:1_16:1	[M + NH4]+	C_35_H_64_O_5_	DG
16	6.051	612.54742	DG 34:1|DG 16:0_18:1	[M + NH4]+	C_37_H_70_O_5_	DG
17	5.214	608.51532	DG 34:3|DG 16:1_18:2	[M + NH4]+	C_37_H_66_O_5_	DG
18	4.898	606.49695	DG 34:4|DG 16:1_18:3	[M + NH4]+	C_37_H_64_O_5_	DG
19	6.603	640.58044	DG 36:1|DG 18:0_18:1	[M + NH4]+	C_39_H_74_O_5_	DG
20	6.115	638.56628	DG 36:2|DG 18:1_18:1	[M + NH4]+	C_39_H_72_O_5_	DG
21	5.705	636.54767	DG 36:3|DG 18:1_18:2	[M + NH4]+	C_39_H_70_O_5_	DG
22	5.322	634.53503	DG 36:4|DG 18:2_18:2	[M + NH4]+	C_39_H_68_O_5_	DG
23	5.001	632.51526	DG 36:5|DG 18:2_18:3	[M + NH4]+	C_39_H_66_O_5_	DG
24	7.845	722.65991	DG 42:2|DG 24:0_18:2	[M + NH4]+	C_45_H_84_O_5_	DG
25	7.152	596.55109	DG O–34:2|DG O–17:0_17:2	[M + NH4]+	C_37_H_70_O_4_	EtherDG
26	7.219	622.56659	DG O–36:3|DG O–19:1_17:2	[M + NH4]+	C_39_H_72_O_4_	EtherDG
27	6.754	620.55212	DG O–36:4|DG O–19:2_17:2	[M + NH4]+	C_39_H_70_O_4_	EtherDG
28	6.414	628.53979	TG 34:0|TG 10:0_12:0_12:0	[M + NH4]+	C_37_H_70_O_6_	TG
29	6.93	656.57776	TG 36:0|TG 10:0_12:0_14:0	[M + NH4]+	C_39_H_74_O_6_	TG
30	7.455	684.60754	TG 38:0|TG 8:0_14:0_16:0	[M + NH4]+	C_41_H_78_O_6_	TG
31	7.044	682.59131	TG 38:1|TG 10:0_10:0_18:1	[M + NH4]+	C_41_H_76_O_6_	TG
32	7.985	712.63965	TG 40:0|TG 10:0_14:0_16:0	[M + NH4]+	C_43_H_82_O_6_	TG
33	7.559	710.62244	TG 40:1|TG 10:0_12:0_18:1	[M + NH4]+	C_43_H_80_O_6_	TG
34	8.504	740.67419	TG 42:0|TG 10:0_16:0_16:0	[M + NH4]+	C_45_H_86_O_6_	TG
35	8.066	738.65637	TG 42:1|TG 8:0_16:0_18:1	[M + NH4]+	C_45_H_84_O_6_	TG
36	9.019	768.7016	TG 44:0|TG 12:0_14:0_18:0	[M + NH4]+	C_47_H_90_O_6_	TG
37	8.561	766.68695	TG 44:1|TG 10:0_16:0_18:1	[M + NH4]+	C_47_H_88_O_6_	TG
38	8.122	764.67175	TG 44:2|TG 10:0_16:1_18:1	[M + NH4]+	C_47_H_86_O_6_	TG
39	9.469	796.7312	TG 46:0|TG 14:0_16:0_16:0	[M + NH4]+	C_49_H_94_O_6_	TG
40	9.052	794.71594	TG 46:1|TG 14:0_16:0_16:1/TG 12:0_16:0_18:1	[M + NH4]+	C_49_H_92_O_6_	TG
41	8.611	792.70563	TG 46:2|TG 14:0_16:1_16:1/TG 12:0_16:1_18:1/TG 14:0_14:1_18:1	[M + NH4]+	C_49_H_90_O_6_	TG
42	8.191	790.68896	TG 46:3|TG 12:0_16:1_18:2	[M + NH4]+	C_49_H_88_O_6_	TG
43	9.675	810.75385	TG 47:0|TG 15:0_16:0_16:0/TG 15:0_15:0_17:0	[M + NH4]+	C_50_H_96_O_6_	TG
44	9.351	808.73602	TG 47:1|TG 16:0_16:0_15:1	[M + NH4]+	C_50_H_94_O_6_	TG
45	8.953	806.71729	TG 47:2|TG 16:0_15:1_16:1	[M + NH4]+	C_50_H_92_O_6_	TG
46	8.504	804.70636	TG 47:3|TG 15:1_16:1_16:1	[M + NH4]+	C_50_H_90_O_6_	TG
47	9.884	824.7702	TG 48:0|TG 16:0_16:0_16:0	[M + NH4]+	C_51_H_98_O_6_	TG
48	9.483	822.75378	TG 48:1|TG 16:0_16:0_16:1	[M + NH4]+	C_51_H_96_O_6_	TG
49	9.076	820.74121	TG 48:2|TG 16:0_16:1_16:1	[M + NH4]+	C_51_H_94_O_6_	TG
50	8.642	818.7251	TG 48:3|TG 16:1_16:1_16:1	[M + NH4]+	C_51_H_92_O_6_	TG
51	8.279	816.70477	TG 48:4|TG 16:1_16:1_16:2/TG 14:1_16:1_18:2	[M + NH4]+	C_51_H_90_O_6_	TG
52	10.091	838.7887	TG 49:0|TG 16:0_16:0_17:0	[M + NH4]+	C_52_H_100_O_6_	TG
53	9.712	836.7666	TG 49:1|TG 16:0_16:0_17:1/TG 15:0_16:0_18:1	[M + NH4]+	C_52_H_98_O_6_	TG
54	9.019	832.73651	TG 49:3|TG 15:1_17:1_17:1/TG 16:0_16:1_17:2	[M + NH4]+	C_52_H_94_O_6_	TG
55	8.592	830.72235	TG 49:4|TG 15:1_16:1_18:2	[M + NH4]+	C_52_H_92_O_6_	TG
56	10.323	852.8009	TG 50:0|TG 16:0_16:0_18:0	[M + NH4]+	C_53_H_102_O_6_	TG
57	9.918	850.7876	TG 50:1|TG 16:0_16:0_18:1	[M + NH4]+	C_53_H_100_O_6_	TG
58	9.53	848.77069	TG 50:2|TG 16:0_16:1_18:1	[M + NH4]+	C_53_H_98_O_6_	TG
59	9.138	846.75555	TG 50:3|TG 16:0_16:1_18:2	[M + NH4]+	C_53_H_96_O_6_	TG
60	8.741	844.74176	TG 50:4|TG 16:1_16:1_18:2	[M + NH4]+	C_53_H_94_O_6_	TG
61	8.348	842.72314	TG 50:5|TG 16:1_16:1_18:3	[M + NH4]+	C_53_H_92_O_6_	TG
62	10.126	864.80042	TG 51:1|TG 16:0_17:0_18:1	[M + NH4]+	C_54_H_102_O_6_	TG
63	9.751	862.78601	TG 51:2|TG 16:0_17:1_18:1	[M + NH4]+	C_54_H_100_O_6_	TG
64	9.387	860.76953	TG 51:3|TG 16:0_17:1_18:2/TG 15:1_18:1_18:1	[M + NH4]+	C_54_H_98_O_6_	TG
65	9.061	858.75525	TG 51:4|TG 15:1_18:1_18:2	[M + NH4]+	C_54_H_96_O_6_	TG
66	10.709	880.83392	TG 52:0|TG 16:0_16:0_20:0/TG 16:0_18:0_18:0	[M + NH4]+	C_55_H_106_O_6_	TG
67	10.351	878.82001	TG 52:1|TG 16:0_18:0_18:1	[M + NH4]+	C_55_H_104_O_6_	TG
68	9.955	876.80646	TG 52:2|TG 16:0_18:1_18:1	[M + NH4]+	C_55_H_102_O_6_	TG
69	9.595	874.78833	TG 52:3|TG 16:0_18:1_18:2	[M + NH4]+	C_55_H_100_O_6_	TG
70	9.213	872.77362	TG 52:4|TG 16:1_18:1_18:2	[M + NH4]+	C_55_H_98_O_6_	TG
71	8.825	870.75818	TG 52:5|TG 16:1_18:2_18:2	[M + NH4]+	C_55_H_96_O_6_	TG
72	8.441	868.74011	TG 52:6|TG 16:1_18:2_18:3	[M + NH4]+	C_55_H_94_O_6_	TG
73	8.068	866.72589	TG 52:7|TG 16:1_18:3_18:3	[M + NH4]+	C_55_H_92_O_6_	TG
74	9.782	888.80188	TG 53:3|TG 17:1_18:1_18:1	[M + NH4]+	C_56_H_102_O_6_	TG
75	9.435	886.78467	TG 53:4|TG 17:1_18:1_18:2	[M + NH4]+	C_56_H_100_O_6_	TG
76	9.086	884.77094	TG 53:5|TG 17:1_18:2_18:2	[M + NH4]+	C_56_H_98_O_6_	TG
77	11.076	908.86023	TG 54:0|TG 16:0_16:0_22:0	[M + NH4]+	C_57_H_110_O_6_	TG
78	10.718	906.85022	TG 54:1|TG 16:0_20:0_18:1	[M + NH4]+	C_57_H_108_O_6_	TG
79	10.368	904.83752	TG 54:2|TG 18:0_18:1_18:1	[M + NH4]+	C_57_H_106_O_6_	TG
80	9.997	902.82233	TG 54:3|TG 18:1_18:1_18:1	[M + NH4]+	C_57_H_104_O_6_	TG
81	9.637	900.80609	TG 54:4|TG 18:1_18:1_18:2	[M + NH4]+	C_57_H_102_O_6_	TG
82	9.275	898.79083	TG 54:5|TG 18:1_18:2_18:2	[M + NH4]+	C_57_H_100_O_6_	TG
83	8.902	896.77527	TG 54:6|TG 18:2_18:2_18:2	[M + NH4]+	C_57_H_98_O_6_	TG
84	8.536	894.75952	TG 54:7|TG 18:2_18:2_18:3	[M + NH4]+	C_57_H_96_O_6_	TG
85	8.154	892.74481	TG 54:8|TG 18:2_18:3_18:3	[M + NH4]+	C_57_H_94_O_6_	TG
86	7.782	890.72479	TG 54:9|TG 18:3_18:3_18:3	[M + NH4]+	C_57_H_92_O_6_	TG
87	11.417	936.89081	TG 56:0|TG 16:0_16:0_24:0	[M + NH4]+	C_59_H_114_O_6_	TG
88	11.088	934.8833	TG 56:1|TG 16:0_22:0_18:1	[M + NH4]+	C_59_H_112_O_6_	TG
89	10.757	932.86841	TG 56:2|TG 20:0_18:1_18:1/TG 22:0_16:1_18:1	[M + NH4]+	C_59_H_110_O_6_	TG
90	10.391	930.84924	TG 56:3|TG 18:1_18:1_20:1	[M + NH4]+	C_59_H_108_O_6_	TG
91	10.058	928.83551	TG 56:4|TG 18:1_20:1_18:2	[M + NH4]+	C_59_H_106_O_6_	TG
92	9.693	926.82471	TG 56:5|TG 20:1_18:2_18:2	[M + NH4]+	C_59_H_104_O_6_	TG
93	11.417	962.91492	TG 58:1|TG 16:0_24:0_18:1	[M + NH4]+	C_61_H_116_O_6_	TG
94	11.114	960.90045	TG 58:2|TG 24:0_16:1_18:1/TG 22:0_18:1_18:1	[M + NH4]+	C_61_H_114_O_6_	TG
95	10.802	958.88574	TG 58:3|TG 24:0_16:1_18:2/TG 22:0_18:1_18:2	[M + NH4]+	C_61_H_112_O_6_	TG
96	10.483	956.87036	TG 58:4|TG 22:0_18:2_18:2	[M + NH4]+	C_61_H_110_O_6_	TG
97	11.592	976.92822	TG 59:1|TG 16:0_25:0_18:1	[M + NH4]+	C_62_H_118_O_6_	TG
98	11.283	974.91675	TG 59:2|TG 25:0_16:1_18:1	[M + NH4]+	C_62_H_116_O_6_	TG
99	10.991	972.8988	TG 59:3|TG 23:0_18:1_18:2/TG 25:0_16:1_18:2	[M + NH4]+	C_62_H_114_O_6_	TG
100	11.748	990.95013	TG 60:1|TG 16:0_26:0_18:1	[M + NH4]+	C_63_H_120_O_6_	TG
101	11.449	988.93219	TG 60:2|TG 24:0_18:1_18:1/TG 26:0_16:1_18:1	[M + NH4]+	C_63_H_118_O_6_	TG
102	11.153	986.9165	TG 60:3|TG 24:0_18:1_18:2/TG 26:0_16:1_18:2	[M + NH4]+	C_63_H_116_O_6_	TG
103	10.854	984.90588	TG 60:4|TG 24:0_18:2_18:2	[M + NH4]+	C_63_H_114_O_6_	TG
104	11.895	1004.96466	TG 61:1|TG 16:0_27:0_18:1	[M + NH4]+	C_64_H_122_O_6_	TG
105	11.605	1002.95099	TG 61:2|TG 25:0_18:1_18:1/TG 27:0_16:1_18:1	[M + NH4]+	C_64_H_120_O_6_	TG
106	11.322	1000.92993	TG 61:3|TG 25:0_18:1_18:2	[M + NH4]+	C_64_H_118_O_6_	TG
107	11.036	998.91034	TG 61:4|TG 25:0_18:2_18:2	[M + NH4]+	C_64_H_116_O_6_	TG
108	12.054	1018.98224	TG 62:1|TG 16:0_28:0_18:1	[M + NH4]+	C_65_H_124_O_6_	TG
109	11.761	1016.96429	TG 62:2|TG 26:0_18:1_18:1	[M + NH4]+	C_65_H_122_O_6_	TG
110	11.488	1014.94641	TG 62:3|TG 26:0_18:1_18:2	[M + NH4]+	C_65_H_120_O_6_	TG
111	11.205	1012.92883	TG 62:4|TG 26:0_18:2_18:2	[M + NH4]+	C_65_H_118_O_6_	TG
112	11.914	1030.98181	TG 63:2|TG 27:0_18:1_18:1	[M + NH4]+	C_66_H_124_O_6_	TG
113	11.65	1028.96179	TG 63:3|TG 27:0_18:1_18:2	[M + NH4]+	C_66_H_122_O_6_	TG
114	12.073	1045.00183	TG 64:2|TG 28:0_18:1_18:1	[M + NH4]+	C_67_H_126_O_6_	TG
115	11.802	1042.98071	TG 64:3|TG 28:0_18:1_18:2	[M + NH4]+	C_67_H_124_O_6_	TG
116	8.217	864.76392	TG 50:2;1O|TG 16:0_18:1_16:1;1O	[M + NH4]+	C_53_H_98_O_7_	OxTG
117	7.791	862.74622	TG 50:3;1O|TG 16:0_18:2_16:1;1O	[M + NH4]+	C_53_H_96_O_7_	OxTG
118	8.726	892.79755	TG 52:2;1O|TG 16:0_18:1_18:1;1O	[M + NH4]+	C_55_H_102_O_7_	OxTG
119	8.282	890.77795	TG 52:3;1O|TG 16:0_18:1_18:2;1O	[M + NH4]+	C_55_H_100_O_7_	OxTG
120	7.479	880.76093	TG 50:2;2O|TG 17:1_17:1_16:0;2O	[M + NH4]+	C_53_H_98_O_8_	OxTG
121	7.086	878.7486	TG 50:3;2O|TG 16:0_19:2_15:1;2O	[M + NH4]+	C_53_H_96_O_8_	OxTG
122	6.661	876.73419	TG 50:4;2O|TG 16:1_16:1_18:2;2O	[M + NH4]+	C_53_H_94_O_8_	OxTG
123	7.923	908.79285	TG 52:2;2O|TG 16:0_19:1_17:1;2O	[M + NH4]+	C_55_H_102_O_8_	OxTG
124	7.571	906.77588	TG 52:3;2O|TG 18:1_19:2_15:0;2O	[M + NH4]+	C_55_H_100_O_8_	OxTG
125	7.17	904.76459	TG 52:4;2O|TG 16:0_18:2_18:2;2O	[M + NH4]+	C_55_H_98_O_8_	OxTG
126	6.794	902.74146	TG 52:5;2O|TG 18:2_19:2_15:1;2O	[M + NH4]+	C_55_H_96_O_8_	OxTG
127	7.973	934.80988	TG 54:3;2O|TG 18:1_18:1_18:1;2O	[M + NH4]+	C_57_H_104_O_8_	OxTG
128	7.633	932.79602	TG 54:4;2O|TG 18:1_18:1_18:2;2O	[M + NH4]+	C_57_H_102_O_8_	OxTG
129	7.278	930.77985	TG 54:5;2O|TG 18:2_19:2_17:1;2O	[M + NH4]+	C_57_H_100_O_8_	OxTG
130	10.873	1103.00757	TG 66:3;O2|TG 16:1_18:1_16:0;O(FA 16:0)	[M + NH4]+	C_69_H_128_O_8_	TG_EST
131	11.174	1131.03638	TG 68:3;O2|TG 18:1_18:1_16:0;O(FA 16:0)	[M + NH4]+	C_71_H_132_O_8_	TG_EST
132	10.912	1129.01904	TG 68:4;O2|TG 18:1_18:1_16:0;O(FA 16:1)	[M + NH4]+	C_71_H_130_O_8_	TG_EST
133	9.976	876.83447	TG O–53:2|TG O–17:0_18:1_18:1/TG O–19:1_16:0_18:1	[M + NH4]+	C_56_H_106_O_5_	EtherTG
134	9.249	898.82886	TG O–55:5|TG O–19:1_18:2_18:2/TG O–19:2_18:1_18:2	[M + NH4]+	C_58_H_104_O_5_	EtherTG

## Data Availability

Data are contained within the article.
